# Exploring Risk Factors Associated With Early Mother's Own Milk Feeding Cessation in Very Low Birth Weight Infants

**DOI:** 10.1111/mcn.70057

**Published:** 2025-06-15

**Authors:** Kaan Karacan, Nadine Scholten, Isabella Schwab, Tim Ohnhäuser, Till Dresbach, Neo‐MILK, Nadine Scholten, Andreas Müller, Till Dresbach, Martin Hellmich, Nicole Ernstmann, Antje Hammer, Friederike Eyssel, Angela Kribs, Juliane Köberlein‐Neu, Katharina Lugani, Eva Mildenberger, Jens Ulrich Rüffer, Katja Matthias, Anne Sunder‐Plaßmann, Daniel Wiesen, Dirk Horenkamp‐Sonntag, Iris Klein, Melanie Klein, Christoph Rupprecht, Laura Schleich, Olaf Beckmann, Anke Kurz

**Affiliations:** ^1^ Institute of Medical Sociology, Health Services Research, and Rehabilitation Science University of Cologne Cologne Germany; ^2^ Department of Neonatology and Pediatric Intensive Care, Children's Hospital University of Bonn Bonn Germany; ^3^ IMVR University of Cologne, University Hospital Cologne; ^4^ University Hospital Bonn; ^5^ IMSB University Hospital Cologne; ^6^ IfPS University Hospital Bonn; ^7^ CITEC University Bielefeld; ^8^ University Hospital Cologne; ^9^ Bergisches Competence Centre for Health Economics and Health Services Research University Wuppertal; ^10^ Medical Law University Düsseldorf; ^11^ University Hospital Mainz; ^12^ TAKEPART Media+Science GmbH; ^13^ Frauenmilchbank‐Initiative; ^14^ Behavioral Management Science University of Cologne; ^15^ Techniker Krankenkasse; ^16^ DAK‐Gesundheit; ^17^ AOK Rheinland/Hamburg; ^18^ Pronova BKK

**Keywords:** breast milk expression, breastfeeding, human milk, infant, lactation, survival analysis, very low birth weight

## Abstract

Infants born with a very low birth weight (VLBW, < = 1.500 g) have an increased risk for medical complications and long‐term impairments. Feeding these infants with their mother's own milk (MOM) reduces the risk for adverse outcomes, but many VLBW infants are not fed with MOM for the recommended duration of at least 6 months postpartum. This study examines factors associated with early cessation during the VLBW infants' neonatal intensive care unit (NICU) stay and after discharge. Data were collected from an anonymous, nationwide survey as part of the Neo‐MILK study. Logistic regressions and Cox proportional hazard models were used to identify factors associated with early cessation of MOM feeding. Among the 304 mothers analysed, 19.4% of all mothers ceased MOM feeding during the infants' NICU stay. The total cessation rate before 6 months was 53.9%. An early milk volume of over 500 mL/day compared to less or equal to 500 mL/day was negatively associated with MOM feeding cessation during the infants' NICU stay (Adjusted OR: 0.14). Exclusive pumping was associated with a higher cessation rate after discharge (Adjusted HR: 2.01). Early sufficient milk volume and mixed feeding (pumping and breastfeeding) inform longer MOM feeding duration. Interventions targeting early lactation practices and promoting direct breastfeeding while helping with the transition from pumping to breastfeeding are essential for improving MOM feeding outcomes in VLBW infants.

**Trial Registration**: German Register of Clinical Trials, ID: DRKS00024799, https://drks.de/search/en/trial/DRKS00024799.

## Summary

- Professional lactation support is vital during the NICU stay, especially for mothers of VLBW infants who struggle to reach a milk volume of 500 mL/day by Day 14 postpartum.
- Exclusive pumping is associated with a higher risk of early cessation, highlighting the need to promote a combination of pumping and breastfeeding while supporting the mothers during the transition to direct breastfeeding.
- Given the unique challenges faced by mothers of VLBW infants, focused interventions are essential to extend MOM feeding durations and thus improve health outcomes for both mother and infant.

## Graphical abstract

Early cessation of mother's own milk (MOM) feeding in very low birth weight infants is strongly linked to low milk volume by Day 14 postpartum and exclusive pumping after discharge. Promoting early lactation support and mixed feeding (pumping and breastfeeding) strategies may help extend MOM feeding duration.

**Abbreviations**
HR: hazard ratio
MOM: mother's own milk
NICU: neonatal intensive care unit
OR: odds ratio
VLBW: very low birth weight

## 1 Introduction

Infants born with a very low birth weight (VLBW, < = 1.500 g) have an increased risk of experiencing complications such as necrotizing enterocolitis, retinopathy of prematurity and late‐onset sepsis, as well as long‐term impairments (DiBiasie 2006; Hornik et al. 2012; Latal‐Hajnal et al. 2003; Uauy et al. 1991). Feeding these vulnerable infants with their mother's own milk (MOM) greatly reduces the risk for adverse outcomes (Dutta et al. 2015; M. G. Parker et al. 2021). The World Health Organization (WHO) strongly recommends the feeding with MOM for the first 6 months after birth for all newborns (WHO 2022). Even though VLBW infants are the ones that would gain the most from MOM feeding, many of these infants are not fed with MOM for the recommended minimum duration (Bonnet et al. 2019; Casavant et al. 2015; Chauhan et al. 2022). Contributing factors are the difficulty of initiating breastfeeding due to frequent separation of mother and infant as well as pumping dependence due to the infant's immaturity (Cregan et al. 2002; Geddes et al. 2013).

A milk volume of over 500 mL/day on Day 14 postpartum is referred to as coming to volume and indicates a sufficient milk supply for exclusive MOM feeding (Meier et al. 2010). It has also been shown that early milk supply informs later milk volume (L. A. Parker et al. 2012). However, many mothers of VLBW infants struggle to come to volume (Hill et al. 2005; Hoban et al. 2018) and insufficient MOM production is often cited by mothers as a reason for discontinuing MOM feeding (M. G. Parker et al. 2021). The neonatal intensive care unit (NICU) staff is of particular relevance here, as their attitude and support can significantly influence the mother's lactation success (M. G. Parker et al. 2021). Another challenge for prolonged MOM feeding duration in pump‐dependent mothers is the transition from pumping to breastfeeding. Existing studies on MOM feeding duration and mode of feeding present mixed results, with some suggesting that exclusive pumping is linked to a prolonged duration in mature newborns (Meehan et al. 2008; Win et al. 2006), while others indicate that breastfeeding is associated with a longer duration (Keim et al. 2017; Yourkavitch et al. 2018). Yet, sustained feeding through milk expression has been linked to a decrease in milk volume (Hill et al. 2007) and qualitative research has highlighted the perceived time‐consuming and inconvenient nature of pumping for many mothers (Felice et al. 2017; Madiba and Sengane 2021).

Studies, mostly about term infants, found that demographics (education, maternal age, ethnicity) (Bertini et al. 2003; Evers et al. 1998; Jones et al. 2015; Milligan et al. 2000; Thulier and Mercer 2009) and infant‐related factors (gestational age) (Gatti 2008; Jonsdottir et al. 2021; Meedya et al. 2010) have a significant influence on breastfeeding duration. In addition, gestational age has been shown to predict coming to volume among mothers of VLBW infants as prematurity can lead to delays in the onset of MOM production (Hoban et al. 2018). The early inability to breastfeed due to impairments in the VLBW infants inform challenges surrounding the transition to breastfeeding (Jiang and Jiang 2022; M. G. Parker et al. 2021). Maternal education, maternal age and ethnicity have been shown to be associated with the establishment of breastfeeding (Pineda 2011; Zachariassen et al. 2010), while older maternal age has also been shown to be a predictor of delays in lactogenesis (Dong et al. 2022).

We aim to take a deeper look into MOM feeding durations among mothers of VLBW infants. This group has been underrepresented in prior research, even though VLBW infants profit immensely from MOM feeding. Given that hospital staff interventions might prevent early weaning during the NICU stay, we analyse it separately from weaning at home. A milk volume of over 500 mL/day by Day 14 postpartum has been associated with continued MOM feeding after discharge (Hoban et al. 2018). We hypothesize that in our sample, there is a positive association between achieving a milk volume of more than 500 mL/day and not ceasing to feed MOM during the NICU stay. Further, we expect that exclusive pumping after discharge will be negatively associated with a prolonged MOM feeding duration in VLBW infants. Compared to the demands of exclusive pumping, direct breastfeeding paired with pumping might be more sustainable for these mothers in the long term. We define mixed feeding as the combination of breastfeeding and feeding any pumped MOM. The additional feeding of non‐MOM alternatives is not relevant to the purpose of this study. Other factors associated with breastfeeding duration, namely gestational age, maternal education, migratory biography and maternal age, will be considered as potential confounders to ensure the accuracy and validity of the results.

## 2 Methods

The data analysed here were obtained from an anonymous, nationwide paper‐based survey conducted as a part of the Neo‐MILK study (Scholten et al. 2023). This project is funded by the Innovation Fund of the Federal Joint Committee (funding code: 01NVF19027) and registered in the German Register of Clinical Trials (ID: DRKS00024799). To determine the status quo of care in hospitals from the mothers' perspective, a survey was conducted in Germany from June to August 2021 in collaboration with four statutory health insurance companies (AOK Rhineland/Hamburg, TK, DAK, Pronova BKK). Only mothers who gave birth to infants with a birthweight of < = 1.500 g (ICD‐10 criteria: P07.00, P07.01, P07.02, P07.10 and P07.11) in the past 6–24 months at the time of data collection were invited to participate in the survey. The health insurance companies reminded the mothers once to participate using a combined thank you and reminder letter. The survey package included a small band‐aid set, an emergency poster and a postcard which the participants could fill out to participate in a separate lottery for shopping vouchers. These incentives were unconditional as the study participation was not mandatory. The completed questionnaires were sent directly to the study group by the mothers, without any involvement from the health insurance companies. The survey was approved by the Ethics Committee of the University Hospital of Cologne (ethics committee vote: 20‐1547). The questionnaire can be provided upon request.

The outcome variable, MOM feeding duration, was measured by asking the mothers how long they pumped for or breastfed their infant in months and weeks. The duration was converted to weeks by multiplying the number of months by 4.345 (average length of a month in weeks) and adding the rounded number to the remaining weeks to receive a close approximation of the total weeks. The threshold of interest is 6 months which translates to almost exactly 26 weeks, thereby avoiding any calculation and interpretation issues by using weeks instead of months. Given that infants had to be at least 6 months old at the time of the survey, any duration beyond this point was deemed incomparable and all values exceeding 26 weeks were recoded to 26 weeks.

Mothers were asked about their milk volume on day 14 postpartum. Over 500 mL/day is widely regarded as a sufficient milk volume (Mago‐Shah et al. 2023). Therefore, value zero represents a milk supply of under or equal to 500 mL/day and value one a milk supply of over 500 mL/day. To record the transition from pumping to breastfeeding, the question was asked whether only pumping (1) or breastfeeding and pumping (0) had taken place up to the time of the survey.

Various confounders were included to account for influences that might skew the results. Educational degrees were categorized into three levels based on the International Standard Classification of Education 2011 (ISCED 2011) for cross‐national comparability (OECD, European Union, UNESCO Institute for Statistics 2012). Some ISCED 2011 levels were combined because the data set was quite homogeneous in terms of educational attainment. The first level (0) includes all respondents without formal degrees and those with lower secondary education, the second level (1) comprises upper secondary education, and the highest level (2) includes individuals with university and applied science degrees. First language is used as a proxy for migratory biography and was operationalized as “German” (1) and “Other languages” (0) due to the diverse range of first languages among nonnative German speakers.

Descriptive characteristics are presented as both frequencies and percentages. The variables are assessed through descriptive methods (percentages, means, correlation tests and Kaplan–Meier survival curves) and univariable methods (log‐rank test and unadjusted hazard ratio). Logistic regressions (cessation during infants NICU stay) and Cox proportional hazard regressions (MOM feeding duration after discharge) are used to identify associated factors. In the survival time analysis, Kaplan–Meier plots and log‐rank tests are used for binary and categorical variables. The log‐rank test is employed to assess whether significant differences exist between survival curves. Predictors with a *p*‐value of 0.25 or lower are recommended for inclusion in the multivariable Cox regression model, as higher *p*‐values suggest minimal contribution to the model. The continuous predictor, maternal age, is evaluated using an unadjusted Cox regression. These initial analyses aim to identify variables unlikely to influence the outcome, which are then subsequently excluded. The remaining predictors are included in adjusted Cox proportional hazard regression models. Additionally, the proportionality assumption is assessed for each predictor, and model comparison is performed by using Akaike Information Criterion (AIC) and Bayesian Information Criterion (BIC). Model fit is evaluated using Cox‐Snell residuals. Stata 18.0 is used for the analysis.

## 3 Results

**Figure 1:**
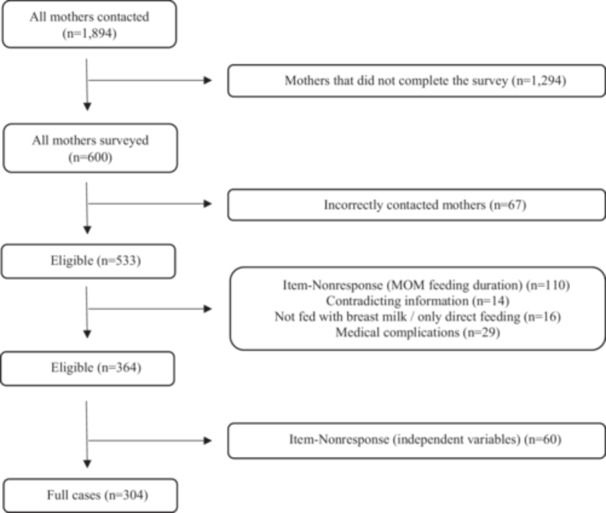
Flow chart of the sample restriction.

In total, 1894 mothers were initially contacted, of which 600 returned the completed questionnaire, resulting in a response rate of 31.67%. 67 mothers were incorrectly contacted as they did not meet the ICD‐10 inclusion criteria and were subsequently excluded. 110 cases were excluded from this analysis due to unavailable information on MOM feeding duration. Since no auxiliary variables were identified and the data are missing completely at random, we did not perform multiple imputation (Jakobsen et al. 2017). Mothers who fed MOM longer to the infant than the infant in question was old, did not feed MOM at all, and/or fed exclusively at the breast for the entire duration were excluded. Additionally, mothers who reported discontinuing breastfeeding due to medical complications in their infant were excluded, as this is considered an infant‐related reason for early cessation of breastfeeding. Missing data are present in at least one of the independent variables for 60 observations. Following Rubin's classification (Rubin 1976), it is assumed that the data is missing at random after thorough examination of the patterns in the data. This resulted in a final sample size of 304 complete cases. Regarding clustering, no significant clustering issues were identified, as the participants were not grouped by any specific characteristics. Further information can be derived from Figure 1.

During the NICU stay, 19.4% of all mothers in the final sample ceased to pump and/or breastfeed. The average duration infants spent in the NICU was 9.6 weeks (SD: 4.9). Examining the entire duration, three out of 304 mothers ceased MOM feeding within the first week after birth. Including the 13th week (corresponding to approximately 3 months), the cumulative percentage of cessation was 35.2% (95% CI: 0.30–0.41). The cumulative cessation for the first 25 weeks was 53.9% (95% CI: 0.48–0.59), meaning that less than half of all respondents fed their VLBW infants with any MOM for at least 26 weeks (6 months). On average, VLBW infants were fed with MOM for 18.4 weeks (SD: 8.2). Table 1 presents the characteristics of mothers with VLBW infants split by cessation of MOM feeding during NICU stay and after discharge. The mothers were on average around 34 years old with the youngest being 22 and the oldest 49 years old. Throughout the entire feeding period, 53.3% of mothers exclusively fed their infant through pumped milk. Respondents feeding their infant with MOM after discharge had a much higher average milk volume on day 14 postpartum.

**Table 1:** Characteristics of mothers with VLBW infants.

	Ceased MOM feeding during NICU stay (*N* = 59)	Fed MOM after discharge (*N* = 245)	Total (*N* = 304)
Milk volume on day 14 postpartum			
< = 500 mL/day	53 (89.8%)	137 (55.9%)	190 (62.5%)
> 500 mL/day	6 (10.2%)	108 (44.1%)	114 (37.5%)
Mode of feeding			
Mixed feeding	6 (10.2%)	136 (55.5%)	142 (46.7%)
Exclusive pumping	53 (89.8%)	109 (44.5%)	162 (53.3%)
Gestational age			
Extremely preterm (< 28 weeks)	21 (35.6%)	90 (36.7%)	111 (36.5%)
Very preterm (28 to 31 weeks)	30 (50.8%)	109 (44.5%)	139 (45.7%)
Moderate or late preterm (32 to < 37 weeks)	8 (13.6%)	46 (18.8%)	54 (17.8%)
Maternal education			
No formal and lower secondary education	26 (44.1%)	68 (27.8%)	94 (30.9%)
Upper secondary education	13 (22.0%)	70 (28.6%)	83 (27.3%)
University degree (including applied sciences)	20 (33.9%)	107 (43.7%)	127 (41.8%)
Mother language			
Not German	13 (22.0%)	40 (16.3%)	53 (17.4%)
German	46 (78.0%)	205 (83.7%)	251 (82.6%)
Age	34.6 (5.3)	32.9 (4.6)	34.0 (4.7)

The effect of early milk volume on MOM feeding duration during the infants' NICU stay is analysed using logistic regression models in Table 2. Maternal age was not included in these models due to multicollinearity. The unadjusted Odds Ratios (OR) show that mothers who had a milk volume on Day 14 postpartum of over 500 mL/day and mothers with a higher educational attainment were less likely to cease pumping/breastfeeding during their infants' NICU stay. Especially milk volume is strongly associated with weaning before discharge. The effect size is not affected by the inclusion of confounders.

**Table 2:** Factors associated with MOM feeding cessation during hospital stay.

	Unadjusted OR (95% CI)	Adjusted OR (95% CI)
Milk volume on Day 14 postpartum over 500 mL/day	0.14*** (0.06–0.35)	0.14*** (0.06–0.33)
Gestational age^a^		
Very preterm (28 to 31 weeks)	1.18 (0.63–2.20)	1.12 (0.58–2.16)
Moderate or late preterm (32 to < 37 weeks)	0.74 (0.31–1.81)	0.75 (0.30–1.88)
Maternal education^b^		
Upper secondary education	0.49* (0.23–1.02)	0.51* (0.23–1.10)
University degree (including applied sciences)	0.49** (0.25–0.94)	0.51** (0.25–1.02)
German as mother language	0.69 (0.34–1.40)	0.56 (0.27–1.20)
Constant		0.95 (0.38–2.39)

^a^ Reference group: Extremely preterm (< 28 weeks).

^b^ Reference group: No formal and lower secondary education.

***p < 0.001; **p < 0.01; *p < 0.05.

**Figure 2:**
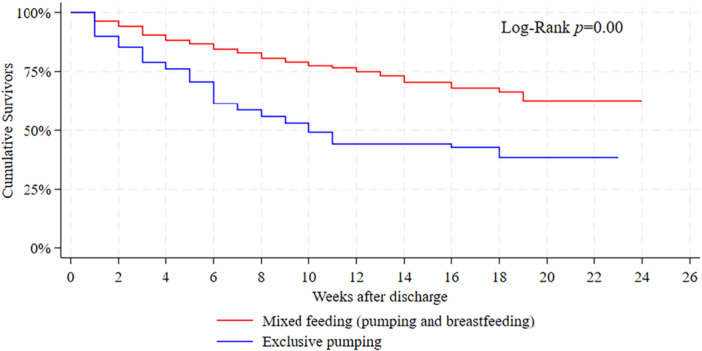
Survival curves of MOM feeding duration by mode of feeding. The red line represents mixed feeding (pumping and breastfeeding) and the blue line represents exclusive pumping.

Figure 2 displays the Kaplan–Meier survival curves of feeding with MOM duration after discharge separated by mode of feeding. The plot reveals that the cumulative number of MOM feeding mothers at any given time is highest among mothers who fed their infants through both pumping and breastfeeding. Specifically, 65.5% of mothers who utilized mixed feeding, and 29% of those who exclusively pumped, fed MOM for at least 6 months postpartum. No variable showed a *p*‐value higher than the cutoff value in the log‐rank test, thereby providing strong statistical evidence of differing survival distributions for all variables. Additionally, age was assessed separately using the unadjusted hazard ratio (HR) and showed a *p*‐value of 0.85. Similar to the variable selection in the logistic model, maternal age will not be considered in the multivariable Cox model. All other variables are likely to contribute to the models, meaning no variable will be forcefully included into any of them.

The multivariable results are presented in Table 3. Changes in the HRs can be examined by comparing the unadjusted and adjusted Cox proportional hazard regressions. The unadjusted HR for exclusive pumping indicates that the rate to cease feeding with MOM increases by approximately 128% for mothers who exclusively pumped compared to those who fed their infants via mixed feeding. Model II presents the results of a Cox regression with the full set of predictors. While the effect size of exclusive pumping decreases in the adjusted model, the effect remains strong and highly significant.

**Table 3:** Effects of mode of feeding and other variables on MOM feeding duration after discharge using univariable (unadjusted) and multivariable (adjusted) Cox proportional hazard regression models.

	Hazard ratio (95% CI)					
	Unadjusted		Adjusted			
	I		II		III^a^	
Exclusive pumping^b^	2.28***	(1.55–3.37)	2.03***	(1.34–3.10)	2.01***	(1.33–3.03)
Milk volume on Day 14 postpartum over 500 mL/day	0.49***	(0.32–0.73)	0.53***	(0.35–0.80)		
Gestational age^c^						
Very preterm (28 to 31 weeks)	1.12	(0.71–1.77)	1.04	(0.66–1.65)		
Moderate or late preterm (32 to < 37 weeks)	1.33	(0.78–2.26)	1.36	(0.80–2.34)		
Maternal education^d^						
Upper secondary education	0.72	(0.45–1.14)	0.87	(0.53–1.41)	0.95	(0.58–1.55)
University degree (including applied sciences)	0.41***	(0.26–0.65)	0.51**	(0.32–0.83)	0.52**	(0.32–0.84)
German as mother language	1.65	(0.92–2.95)	1.45	(0.81–2.61)		
AIC			1069.66		936.07	
BIC			1094.17		946.57	

^a^ Stratified across milk volume on Day 14 postpartum.

^b^ 0 = Mixed feeding (pumping and breastfeeding).

^c^ Reference group: Extremely preterm (< 28 weeks).

^d^ Reference group: No formal and lower secondary education.

***p < 0.001; **p < 0.01.

To assess non‐proportionality, the adjusted model was tested using time‐dependent covariates, meaning interactions with log(time). The milk volume on Day 14 postpartum was highly significant (*p* = 0.00), suggesting non‐proportional hazards. Therefore, it was excluded from the model, and stratification was employed to control for the effect of this variable. Model III presents the stratified Cox proportional hazard regression, allowing different baseline hazards for the early milk volume categories while calculating a single HR for each predictor. In this final model, nonsignificant variables were excluded after checking for interactions, leaving only maternal education. Holding maternal education constant and averaging across the milk volume categories, respondents who exclusively pumped showed an increased rate of MOM feeding cessation of approximately 101% compared to those practicing mixed feeding. Model comparison using AIC and BIC shows that the stratified model is the better model. The Goodness‐of‐fit plot for the stratified model revealed that the hazard function only deviates from the reference line toward the end, which is not uncommon for censored data. These findings suggest that the model adequately fits the data and is appropriate for the analysis.

## 4 Discussion

The study results show that approximately half of all mothers in our sample fed their VLBW infants any MOM for at least 6 months. Around every fifth mother weaned during their infants' stay at the NICU. Associated factors were the milk volume on Day 14 postpartum and educational level. These results align with previous findings (Brownell et al. 2012; Haas et al. 2006; Hoban et al. 2018). As hypothesized, the milk volume on Day 14 postpartum functions as an important indicator for early cessation in VLBW infants during the NICU stay. This is likely linked to the long‐term milk production being determined by the early lactation phase (L. A. Parker et al. 2012). Therefore, interventions should focus on the initial days of lactation, specifically targeting pumping practices to enhance the milk volume.

Regarding the time after discharge, a notably higher proportion achieved a longer MOM feeding duration among those mothers who pumped and breastfed. The adverse effect of exclusive pumping on duration persisted across all Cox proportional hazard models. The milk volume on Day 14 postpartum violated the proportionality assumption and was therefore excluded. The effect of coming to volume in the model was likely to be biased. No conclusion can be made about the relevance of the early milk volume for the time after discharge since there was a statistical need to exclude the variable from the Cox model. The association between mode of feeding and MOM feeding duration after discharge suggests that mixed feeding is of high importance for sustaining MOM feeding in VLBW infants. This result aligns with previous findings (Fan et al. 2022; Pang et al. 2017), although the effect size of the mode of feeding observed in this study seems to be greater. This discrepancy is likely attributable to the unique focus on VLBW infants in this study, a population that has been underrepresented in prior research.

Mothers have been shown to use expressed MOM feeding to avoid problems with breastfeeding (Felice et al. 2017). Despite encouragement to transition to exclusive breastfeeding or mixed feeding (both pumping and breastfeeding), many mothers maintain exclusive pumping (Furman et al. 1998). The present study emphasizes the importance of promoting direct breastfeeding and providing support to mothers navigating the transition during the early postpartum period. To prevent early cessation of breastfeeding, healthcare professionals should provide patient‐centred support tailored to the individual needs of mothers of VLBW infants who do not achieve the necessary milk volume. A focus on maternal experiences and emotional well‐being is crucial for fostering positive breastfeeding experiences and adapting to motherhood (Flacking et al. 2007). Innovative solutions, such as involving lactation consultants, utilizing digital training and support programmes, and promoting interdisciplinary collaboration, are necessary to ensure optimal care for both mothers and infants.

Though comparable studies have been done in other countries (Ericson et al. 2018; Lojander et al. 2024), this is the first study, to our knowledge, that investigated factors influencing weaning during the infants NICU stay and the association between mode of feeding and feeding with MOM cessation after discharge among mothers of VLBW infants in Germany. Surveying mothers when the infants were at least 6 months old enabled us to investigate the MOM feeding duration up until the recommended 6 months.

However, retrospective data collection introduces potential issues such as recall bias. In addition, the data were collected during the COVID‐19 pandemic which could have affected the response rate and results. Limitations of the data set include missing data, and absence of information on other established or potential predictors of MOM feeding duration like, for example, income, occupation, place of residence, obesity and maternal depression. Information about the treating hospital would have enabled further analyses of regional differences and comparisons of breastfeeding rates across hospitals. A more detailed analysis of the distribution of feeding methods (e.g., primarily breastfeeding supplemented with pumped milk and non‐MOM alternatives) would also provide valuable insights. Methodologically, this study focused on associations. Future research utilizing modelling frameworks geared towards causal reasoning could contribute to these findings.

## 5 Conclusion

Weaning during the infant's NICU stay is strongly associated with the milk volume on Day 14 postpartum. Increased support of mothers of VLBW infants not coming to volume should be focused on by healthcare professionals to avoid early cessation, with an emphasis on encouraging mothers that feeding any amount of MOM is beneficial. In comparison to mixed feeding (pumped milk and breastfeeding), exclusive pumping is strongly associated with early cessation after discharge. Therefore, targeted lactation and breastfeeding education as well as supportive measures are essential to help mothers successfully transition to breastfeeding. Healthcare professionals should provide intensive support to mothers and actively promote lactation. This includes practical assistance with breastfeeding and advice on milk production to help prolong the MOM feeding duration.

## Author Contributions

Nadine Scholten conceptualized the study. Nadine Scholten, Isabella Schwab, Tim Ohnhäuser and Till Dresbach designed the survey questionnaire and collected the data. Kaan Karacan prepared and analysed the data. Kaan Karacan drafted and revised the manuscript. Nadine Scholten, Isabella Schwab, Tim Ohnhäuser and Till Dresbach critically reviewed and revised the manuscript.

## Neo‐MILK

The Neo‐MILK collaborators include the following: Prof. Dr. Nadine Scholten, IMVR (University of Cologne, University Hospital Cologne); Prof. Dr. Andreas Müller and Dr. Till Dresbach (University Hospital Bonn); Prof. Dr. Martin Hellmich, IMSB (University Hospital Cologne); Prof. Dr. Nicole Ernstmann, IfPS (University Hospital Bonn); Dr. Antje Hammer, IfPS (University Hospital Bonn); Prof. Dr. Friederike Eyssel, CITEC (University Bielefeld); PD Dr. Angela Kribs (University Hospital Cologne); Prof. Dr. Juliane Köberlein‐Neu (Bergisches Competence Centre for Health Economics and Health Services Research, University Wuppertal); Prof. Dr. Katharina Lugani, Medical Law (University Düsseldorf); Prof. Dr. Eva Mildenberger (University Hospital Mainz); PD Dr. Jens Ulrich Rüffer, Katja Matthias (TAKEPART Media+Science GmbH); Anne Sunder‐Plaßmann, Frauenmilchbank‐Initiative; Prof. Dr. Daniel Wiesen (Behavioral Management Science, University of Cologne); and Dr. Dirk Horenkamp‐Sonntag, Dr. Iris Klein, Techniker Krankenkasse; Dr. Melanie Klein, DAK‐Gesundheit; Christoph Rupprecht, Laura Schleich, Olaf Beckmann, AOK Rheinland/Hamburg; Anke Kurz, Pronova BKK.

## Conflicts of Interest

The authors declare no conflicts of interest.

## Data Availability

The data that support the findings of this study are available from the corresponding author upon reasonable request.
